# Gene Regulatory Network Reconstruction Using Conditional Mutual Information

**DOI:** 10.1155/2008/253894

**Published:** 2008-06-04

**Authors:** Kuo-Ching Liang, Xiaodong Wang

**Affiliations:** 1 Department of Electrical Engineering, Columbia University, New York, NY 10027, USA

## Abstract

The inference of gene regulatory network from expression data is an important area of research that provides insight to the inner workings of a biological system. The relevance-network-based approaches provide a simple and easily-scalable solution to the understanding of interaction between genes. Up until now, most works based on relevance network focus on the discovery of direct regulation using correlation coefficient or mutual information. However, some of the more complicated interactions such as interactive regulation and coregulation are not easily detected. In this work, we propose a relevance network model for gene regulatory network inference which employs both mutual information and conditional mutual information to determine the interactions between genes. For this purpose, we propose a conditional mutual information estimator based on adaptive partitioning which allows us to condition on both discrete and continuous random variables. We provide experimental results that demonstrate that the proposed regulatory network inference algorithm can provide better performance when the target network contains coregulated and interactively regulated genes.

## 1. Introduction

The prediction of the functions of genes and the elucidation of the gene regulatory mechanisms have been an important topic of genomic research. The advances in microarray technology over the past decade have provided a wealth of information by allowing us to observe the expression levels of thousands of genes at once. With the increasing availability of gene expression data, the development of tools that can more accurately predict gene-to-gene interactions and uncover more complex interactions between genes has become an intense area of research.

### 1.1. Background

Gene Clustering Algorithms

Some of the first attempts at determining gene regulations are based on the gene expression clustering algorithms. These algorithms determine genes that are likely to be coregulated by grouping genes that exhibit similar gene expressions under the same conditions. Different clustering algorithms differ in the metric used to measure similarity between gene expressions, and how the metric is used to cluster into groups similarly expressed genes [1]. In [2], a hierarchical clustering algorithm using a correlation coefficient metric is proposed. The K-means algorithm has also been applied to partition genes into different clusters [3]. Other clustering algorithms such as self-organizing map (SOM) [4], mutual-information-based algorithms [5,6], and graph-theory-based algorithms [7] have also been proposed.

Graphical Algorithms

While gene clustering algorithms allow us to discover genes that are coregulated, they do not reveal much of the underlying biological mechanism such as the regulatory pathways. In recent years, many models have been proposed attempting to understand how individual genes interact with each other to govern the diverse biological processes in the cell. In [8–10], gene regulatory network inference based on graphical models is proposed. A graphical model depicts the relationships among nodes in a graph which are considered as random variables. Links between nodes represent dependence of the two variables. For network inference based on the graphical Gaussian model [11,12], the nodes with corresponding random variables  are assumed to be jointly distributed according to the multivariate Gaussian distribution , with mean vector  and covariance matrix . In [13], the gene-to-gene interaction is predicted from expression data using Bayesian networks, another type of graphical model. The dependence relationship between the variables is denoted by a directed acyclic graph where the nodes are associated with the variables , , and the nodes are linked if a dependent relationship exists between the two corresponding variables. Given a set of expression values , the algorithm selects the graph  that best describes  by choosing the graph that maximizes a scoring function based on the Bayes' rule . In [14], gene regulatory network reconstruction based on the dynamic Bayesian network is proposed to support cycles in the network, and time-series data in .

Relevance Network Algorithms

Another method that is related to graphical model is called relevance network. Relevance networks are based on the idea of "covariance graph" where a link exists between genes  and , if and only if the corresponding gene expressions of  and  are marginally dependent [15]. Different measures of dependence have been used in relevance-network-based algorithms. In [16], the correlation coefficient is used to represent the dependence between two genes, and in both [16,17], mutual information is used to measure the nonlinear relationship between the expressions of two genes. Since these metrics are computed from a finite number of samples, a threshold is often imposed so that two nodes are connected if the computed metric between the two nodes is above the threshold. In [17], entropy and joint entropy are first computed based on the histogram, then the mutual information of  and  is computed by . In [18], the proposed ARACNE algorithm uses the Gaussian kernel estimator to estimate the mutual information between the expressions  and  of genes  and . Before estimating  from the observed expressions  and  using the Gaussian kernel estimator,  and  are copula-transformed to take values between 0 and 1. This step is performed so that the expression data are transformed to uniform distribution, and arbitrary artifacts from microarray processing are removed. In gene regulatory networks, if gene  regulates , which in turn regulates , then  and  will also be highly correlated. Using methods based on relevance network, a link will often be incorrectly inferred between  and  due to the high correlation measures. In [18], ARACNE tries to resolve this problem by using the data processing inequality (DPI). From DPI, if , and  form a Markov chain (denoted as ), then [19]. For a triplet of genes where the estimated mutual information of all three pairs of genes exceed the threshold, the link with the lowest mutual information is removed by ARACNE in the DPI step.

While relevance-network-based methods such as ARACNE perform well when the interactions in the gene regulatory network are between pairs of genes, they are unable to completely discover interactions that are results of the joint regulation of the target gene by two or more genes. The XOR interactive regulation is one such interaction that can be recognized only by exploiting the conditional dependence between variables of interest. Using conditional mutual information (CMI), it is possible to detect the XOR and other nonlinear interactive regulation by two genes.

Several recent works have attempted to incorporate information theoretic measures for more than two variables in regulatory network discovery. In [20], a CMI measure where the conditioning variable takes discrete values in two states (high and low) is proposed to discovery the transcriptional interactions in the human B lymphocytes. In [21,22], methods based on both MI and CMI have also been proposed to decrease the false positive rate for the detection of the interactions. In [23], the conditional coexpression model is introduced, and the CMI is used as a measure of conditional coexpression. In [24], an extension of the context likelihood of relatedness (CLR) algorithm [25], called "synergy augmented CLR" is proposed. The technique uses the recently developed information theoretic concept of synergy [26] to define a numerical score for a transcriptional interaction by identifying the most synergistic partner gene for the interaction. In this work, we propose a relevance-network-based gene regulatory network inference algorithm similar to [24], using information theoretic measure to determine the relationship between triplets of genes.

### 1.2. Objective

Here, we make use of both mutual information and conditional mutual information as measures of dependence between gene expressions. The main focus of this work is to discover the potential interactions between genes by adapting the relevance network model, which is also used in [17,18]. The inference of the connectivity, or the "wiring" of the network, is also an important aspect of biological network inference. The proposed network inference algorithm uses an adaptive partitioning scheme to estimate the mutual information between  and  conditioned on , where  can be either discrete or continuous. We show that using both mutual information and conditional mutual information allows us to more accurately detect correlations due to interactive regulation and other complex gene-to-gene relationships. In this work, our primary focus is on the detection of Boolean interactive regulation and other interactions which cause incorrect inferences, such as coregulation and indirect regulation. The experimental results show that the proposed network inference algorithm can successfully detect these types of regulation, and outperform two commonly used algorithms, BANJO and ARACNE.

The remainder of the paper is organized as follows. In Section 2, we present the system model for regulatory network inference. In Section 3, we present the adaptive partitioning algorithms for estimating mutual information and conditional mutual information as well as our proposed network inference algorithm based on MI-CMI. In Section 4, we present experimental results. Section 5 concludes the paper.

## 2. System Model

Suppose that the given set of genes  form a regulatory network, where each node of the network is represented by a gene. Associated with each node,  is a random variable  with unknown steady-state distribution from which the expressions of  are generated. We assume that for gene , we have the vector of  steady-state gene expressions , where  is the gene expression of gene  under condition .

**Figure 1:**
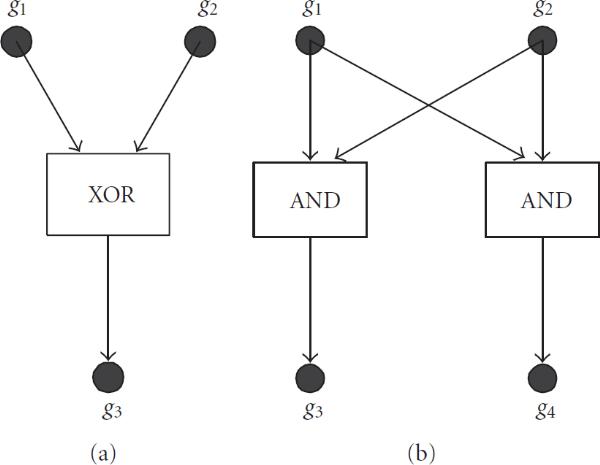
**(a) XOR interactive regulation of  by  and **. (b) Coregulation of  and .

In a network inference problem, our primary goal is to correctly identify the links representing direct regulation and reduce the false negative and false positive links. A false negative can be due to the incorrect estimation of the metric that measures the interaction between the expressions of two genes. When interactive regulation is introduced into the network, false negatives may occur for certain interactive regulations due to that no significant interaction is detected between the regulated gene and any one of the regulating genes, but rather the regulation is only detectable when the regulated gene and all of the regulating genes are considered together. For example, in Figure 1(a), gene  is being regulated by an XOR interaction of genes  and . Using mutual information as metric, the individual interactions between  and  and between  and  are not discovered since .

In the relevance network approach, two nodes are connected when they exhibit high degrees of interaction according to the chosen metric. Using metrics such as correlation coefficient and mutual information, high degrees of interaction between two genes typically indicate that one of the genes is directly or indirectly regulating the other gene, or the two genes are being coregulated by another gene. In relevance networks, indirect regulation and coregulated genes often are the cause of false positive links. ARACNE, as discussed in the previous section, removes indirect regulation by the application of DPI. However, ARACNE and other network inference algorithms based only on correlation coefficient or mutual information are unable to identify genes that are being coregulated, particularly if they are coregulated by the same mechanism. For example, in Figure 1(b), both  and  are regulated by an AND interaction of  and . Using correlation coefficient or mutual information as metric will always result in a high interaction between  and , and in most cases, greater than the interaction between the regulated gene and either one of the regulating genes, whereas using DPI will result in a false positive link.

The insufficiencies of using only mutual information or correlation coefficient as discussed above naturally lead us to the use of conditional mutual information as the metric of choice in our proposed regulatory network inference algorithm. For Figure 1(a), it is clear that the interaction between  and  and that between  and  can be detected by  and . To resolve false positives due to coregulated genes recall that the conditional mutual information  measures the reduction of information provided about  by observing  conditioned on having observed . An example of Figure 1(a) can be seen in [27]. In Figure 1(b), coregulation of  and  can be recognized by the fact that if  and  are regulated by the same biological mechanism,  and , since having observed  or , no more information is provided about  by observing , or information provided about  by observing , respectively. On the other hand, having observed , which regulates both  and , the information provided about  by observing  is reduced, and we have . Thus, by considering both the mutual information and conditional mutual information, we are able to reduce the amount of false positive links due to coregulation. Example of Figure 1(b) can be seen in [28].

From the above discussion, in the next section, we develop a relevance-network-based regulatory network inference algorithm that utilizes both mutual information and conditional mutual information to predict interactions between genes from the observed gene expression data. It is clear that we need efficient estimators that can accurately compute mutual information and conditional mutual information from data. Moreover, the conditional mutual information estimator should be able to support both discrete and continuous conditioning variables to allow for wider ranging uses.

## 3. MI-CMI Regulatory Network Inference Algorithm

There are several mutual information estimators such as the Gaussian kernel estimator and the equipartition estimator [29] but each has its weakness. The Gaussian kernel estimator requires a smoothing window that needs to be optimized for different underlying distributions, thus increasing the estimator complexity. While the equipartition estimator is simple in nature, the different grids in a partition often have variable efficiency in terms of contribution to the mutual information estimate due to the underlying sample distribution. In this section, we make use of an adaptive partitioning mutual information estimator proposed in [30] and extend it to estimating conditional mutual information. These estimators are then employed in building our MI-CMI-based relevance network for regulatory network inference.

### 3.1. Adaptive Partitioning Mutual Information Estimator

Let us consider a pair of random variables  and  taking values in  and , both of which are assumed to be the real line  for simplicity. For each random variable, we have  samples  and . From the samples we wish to obtain an estimate  of the mutual information .

For mutual information estimators that partition the samples according to equal length or equiprobable partition, many of the grids may turn out to be inefficient due to the distribution of the samples. For example, let  and , where  is uniformly distributed on . Hence, the samples fall on a unit circle; and grids inside the circle do not contribute to the estimation of the mutual information between  and . Therefore, a partitioning scheme that can adaptively change the number, size, and placement of the grids is more efficient in estimating mutual information. In the following, we describe a mutual information estimator proposed in [30] that adaptively partitions the observation space based on the unknown underlying distributions of the samples.

In the adaptive partitioning scheme, the sample space  is divided into rectangular grids of varying sizes depending on the underlying distributions. A grid denoted as  has the -axis range  and -axis range . Furthermore, the set containing all the grids of the partitioning is denoted as .

Let us denote , , and  as the densities of the distributions , , and , respectively. We then define the following conditional distributions:(1)

and their densities(2)

respectively, where  denotes the indicator function of the set  can now be written as(3)

where  is called the restricted divergence and  is the residual divergence.

We define a sequence of the partitioning of the sample space  as nested if each grid  is a disjoint union of grids , where  can be different for each . Thus,  can be seen as a refinement of . A nested sequence  is said to be *asymptotically sufficient* for  and  if for every  there exists a  such that for each , one can find an  satisfying(4)

where  denotes the -algebra of , and  denotes the symmetric difference. In [30], it is shown that if the nested sequence  is asymptotically sufficient for  and , then(5)

Given the pairs of samples , we define(6)

that is, the frequency of the samples falling into the grid . Then, the restricted divergence  can be estimated from the samples with the following estimator:(7)

Furthermore, in [30] it is shown that the residual diversity approaches zero as  and that(8)

Thus, mutual information can be estimated by computing the relative sample frequency on appropriately placed rectangular grids.

We now give the adaptive partitioning algorithm that constructs an asymptotic sufficient sequence of partitions for mutual information estimation.

Algorithm 1 (Adaptive partitioning algorithm for mutual information estimation).

(i) Initialization: Partition  and  at  and , respectively, such that(9)

that is,  and  are the equiprobable partition points for  and  with respect to the empirical distribution of marginal distributions, and  is divided into 4 grids. This partition is denoted as .

(ii) Partitioning : for a grid , select the partition points  and , such that(10)

Denote  as the total number of samples in the grid  and  as the total number of samples in each of the quadrants created by the above partition. Compute the Pearson's chi-squared test for uniform distribution,(11)

If the sample distribution of the quadrants passes the uniform test, that is, (11) holds,  is added to . If the sample distribution does not pass the uniform test, the grids , , , and  are added to .

(iii) Repeat step (ii) for all grids in .

(iv) Repeat steps (ii) and (iii) until . When the partitioning process is terminated, define .

(v) Using the partition , compute the mutual information estimate  according to (7).

**Figure 2:**
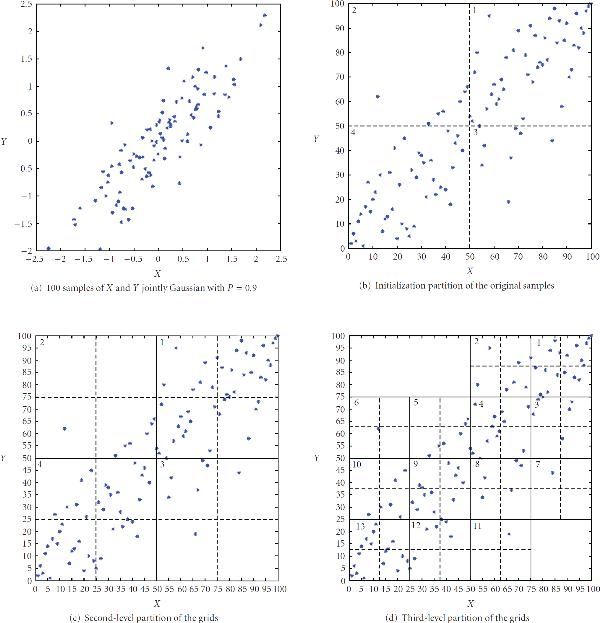
**Example of adaptive partitioning steps for pairwise mutual information**. (a) 100 samples of  and  jointly Gaussian with  (b) Initialization partition of the original samples (c) Second-level partition of the grids (d) Third-level partition of the grids

Here, we give an example of how to adaptively partition a given set of sampled data. In this example, we sampled 100 times  and  that are jointly Gaussian with correlation coefficient of 0.9 and both with mean zero. The 100 sample pairs are plotted in Figure 2(a). In Figure 2(b), we plot the same samples in their ordinal plot, meaning that each sample of  and  is ranked in decreasing order with respect to other samples from the same random variable, and the sample pairs are plotted by their integer-valued ranks. In the ordinal plots, equiprobable partition is equivalent to partition at the midpoint. In Figure 2(b), we can also see the dashed lines dividing the samples into 4 grids. This is the initialization partition that is always kept no matter how the samples are distributed. In Figure 2(c), we can see that the 4 grids are each partitioned into 4 quadrants by the dashed lines. Table 1 shows the distribution of the samples in quadrants created by the partitioning of the 4 grids during the second-level partition, and their chi-squared statistics. To pass the uniform chi-squared test for 95%, the chi-squared test statistic should be less than 7.815. As we can see from Table 1, all 4 grids failed the test, thus require further partitioning.

**Table 1:** Quadrant sample counts in each grid after second-level partition, and result of chi-squared test.

	**Quadrant 1**	**Quadrant 2**	**Quadrant 3**	**Quadrant 4**	** statistic**	**Pass?**
Grid 1	18	7	6	11	8.4762	no
Grid 2	0	0	7	1	17.0000	no
Grid 3	1	6	1	0	11.0000	no
Grid 4	12	6	18	6	9.4286	no

In Figure 2(d), we can see that 13 nonzero grids from the previous steps are each divided into 4 quadrants by the dashed lines. Table 2 shows similarly for the third-level partitions the quadrant sample counts in each of the grids, and their chi-squared test results. From Table 2, we can see that all grids pass the chi-squared test, thus the third-level partition is not needed, and the adaptive partitioning scheme has partitioned the samples into the 13 grids shown in Figure 2(d).

**Table 2:** Quadrant sample counts in each grid after third-level partition, and result of chi-squared test.

	**Quadrant 1**	**Quadrant 2**	**Quadrant 3**	**Quadrant 4**	** statistic**	**Pass?**
Grid 1	7	3	5	3	2.4444	yes
Grid 2	2	1	1	3	1.5714	yes
Grid 3	2	3	0	1	3.3333	yes
Grid 4	3	3	4	1	1.7273	yes
Grid 5	2	0	2	3	2.7143	yes
Grid 6	0	0	1	0	3.0000	yes
Grid 7	0	1	0	0	3.0000	yes
Grid 8	2	2	1	1	0.6667	yes
Grid 9	4	2	5	1	3.3333	yes
Grid 10	2	0	1	3	3.3333	yes
Grid 11	1	0	0	0	3.0000	yes
Grid 12	3	2	1	0	3.3333	yes
Grid 13	2	5	5	6	2.0000	yes

### 3.2. Conditional Mutual Information Estimator

Works in various fields have utilized conditional mutual information to test for conditional independence. However, in most cases, they are often limited to conditioning on a discrete, often binary, random variable [31,32]. When conditioning on a discrete random variable, the conditional mutual information can be computed as(12)

This is done by simply dividing the samples into  bins according to the value  takes, and taking the weighted summation of the mutual information in each bin. In the case of conditioning on a continuous random variable, however, the partitioning of  is often not so clear. Next, we propose a modification to the adaptive partitioning estimator that also adaptively partitions the *z*-axis to allow the estimation of conditional mutual information when the conditioned random variable is continuous.

Let us consider a triplet of random variables , and  taking real values in , and , respectively. Given the samples , , and , we wish to compute an estimate  of the conditional mutual information .

Suppose that the space  is divided into cuboids of various sizes depending on the underlying distributions. The cuboid denoted as  has range  on the -axis,  on the -axis, and  on the -axis, and the set containing all the cuboids of the partition is denoted as . We then define the following conditional distribution:(13)

and its density(14)

Similar to (3), we can write  as(15)

where **Q** denotes  and **R** denotes .

We can rewrite  as(16)

Notice that this is simply a weighted sum for the restricted diversity as computed in (3) for samples grouped according to the *z*-axis partition , and for a partition ,(17)

and it can be estimated as(18)

Following the proof in [30,33],(19)

We can see from (15) and (17) that(20)

and the integral(21)

in the definition of  vanishes if and only if , that is,  and  are independent in the cuboid . In the following, we propose an adaptive partitioning scheme that partitions the given samples into cuboids, where in each cuboid the conditional distributions of  and  given  are independent. Similar to Algorithm 1, we use the Pearson's chi-square test to determine the independence of the samples.

We now present the algorithm for estimating the conditional mutual information with continuous conditioning variable.

Algorithm 2 (Adaptive partitioning algorithm for conditional mutual information estimation).

(i) Initialization: partition , and  at , and , respectively, such that(22)

that is, , and  are the equiprobable partition points for , , and  with respect to the empirical distribution of marginal distributions, and  is divided into 8 cuboids. This partition is denoted as .

(ii) Partitioning : for a cuboid , select the partition points , and , such that(23)

Denote  as the total number of samples in the cuboid  and  as the total number of samples in each of the octants created by the above partition. Compute the Pearson's chi-squared test for uniform distribution,(24)

If the sample distribution passes the uniform test, that is, if (24) holds, the cuboid  is added to . If the sample distribution does not pass the uniform test, the cuboids(25)

are added to .

(iii) Repeat step (ii) for all cuboids in .

(iv) Repeat steps (ii) and (iii) until . When the partitioning process is terminated, define .

(v) Using the partition , compute the conditional mutual information estimate  according to (18).

**Figure 3:**
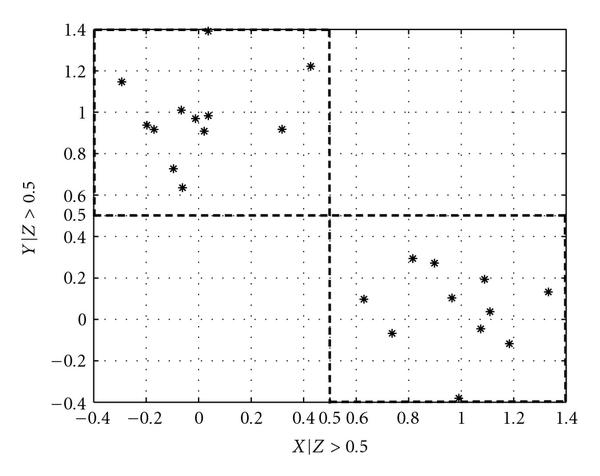
**Adaptive partition of  and  given **.

**Figure 4:**
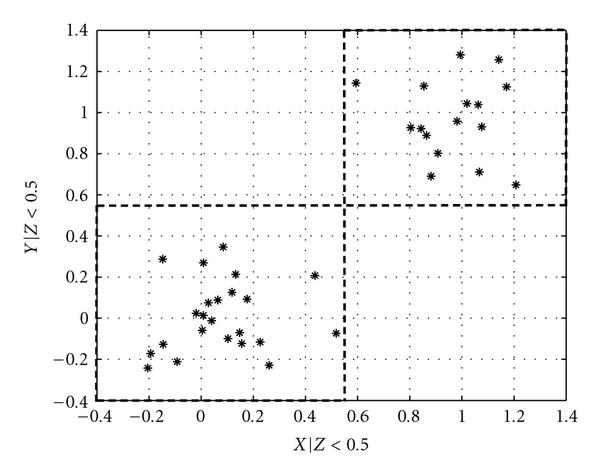
**Adaptive partition of  and  given **.

Figures 3 and 4 give an adaptive partition of a trivariate sample data. Note that  is the output of an XOR gate with  and  as inputs, with random noise added to both the inputs and the output. We can see that the -axis is partitioned into two regions,  and . In the initial step, the sample data is divided into 8 cuboids. The 4 cuboids without any data points are discarded, and the other 4 are added to . In the second step, each of the 4 cuboids is divided into 8 cuboids and tested for uniform distribution with the chi-squared test. All 4 pass the test and are added to . In the next step, we see that , and the partitioning process is terminated with .

Compared to the estimation of conditional mutual information for discrete conditioning variable, we can see that instead of grouping samples into subsets where samples belonging in the same subset have the same values for the discrete-valued conditioning variable, here we group samples based on the adaptively determined partitioning of  on the *z*-axis. The problem of estimating the conditional mutual information is thus broken down into estimating the mutual information for each group of samples, where the samples are grouped by which  they belong to.

Note that the complexity of the Gaussian kernel estimator is known to be . However, the complexity of the adaptive partitioning estimator is dependent upon the joint distribution of the variables. For example, suppose  and  are independent and identically distributed uniform distributions. To compute  from  pairs of  will take on average only the four initializing grids, since the sample pairs are typically uniformly distributed in each of the grids, and no further subpartitions are necessary according to the chi-squared test. On the other hand, suppose that  and , where  is uniformly distributed between , it will take many more subpartitions to obtain uniform distribution of the samples on each of the resulting grids. From our experience, for  samples of  and  jointly Gaussian pairs, the Gaussian kernel estimator takes about 2 minutes to compute the MI, whereas for the adaptive partitioning algorithm, the time is between 2.5 to 3 minutes, on MATLAB code running on a Pentium 4 2.54 GHz machine. However, this is without taking into consideration the overhead required by the Gaussian estimator to compute the smoothing window.

### 3.3. Gene Regulatory Network Inference Algorithm

To infer a gene regulatory network that has various interactive regulations and coregulations, we propose a strategy of using both mutual information and conditional mutual information to reconstruct the regulatory network. In our proposed algorithm, we first use mutual information as metric to build regulatory network similarly to [17] to capture most of the direct regulations. To decrease the complexity of the algorithm by avoiding computing conditional mutual information for all triplets, while still allowing us to detect most of the causes for false positives and false negatives, we only compute the CMI for triplets of genes where either all three genes are connected, or all three genes are not connected. The decrease in complexity would depend on several factors. Once the pairwise MI threshold is chosen, the triplets that have one or two connections between the three genes indicate that the pairwise MI is sufficient for the determination of the interaction between the three genes, and the use of CMI is not necessary. Thus, instead of computing the CMI for all triplets of genes, CMI needs to be computed only for those triplets that are completely connected or completely unconnected. The amount of decrease in complexity would then depend on the ratio of triplets that have only one or two connections, which would depend on the actual connectivities between the genes, and the threshold selected for the pairwise mutual information phase of the algorithm.

The MI-CMI gene regulatory network inference algorithm is as follows.

*Algorithm 3* (MI-CMI gene regulatory network inference algorithm).

(i) For a gene expression dataset containing  genes, compute the mutual information estimate  for all gene pairs , using Algorithm 1.

(ii) Initialize the graph  as a zero matrix. Set  if , where  is a predetermined threshold.

(iii) Detecting indirect regulation and coregulation: for any triplet of genes  where , compute the conditional mutual information estimate , , and  using Algorithm 2.

(a) If(26)

this means that  and  contain nearly the same information regarding , that having observed ,  contains no new information about , and vice versa. Also, having observed , the information contained about  in  is reduced. This indicates that  and  are regulated by  through the same mechanism, meaning that the gene pair  is coregulated, thus  is set to .

(b) If(27)

and , this indicates that  regulates , and  regulates , and that the  is indirectly regulated by , indicated by the smallest CMI. Using DPI similarly to [18],  is set to .

(iv) Detecting interactive regulation: for any triplet of genes  where , compute the conditional mutual information estimate , and  using Algorithm 2.

(a) If one or two of the CMI estimates is greater than , this indicates that the genes contain interactions that was not captured using MI, and we set the corresponding link or links to .

(b) If all three of the CMI estimates are greater than , this may indicate that the two regulating genes may have had some prior interactions, or there is an XOR interaction between the 3 genes. Thus, we apply the DPI to remove the link with the weakest estimated CMI, and the links corresponding to the two largest estimated CMI are set to .

## 4. Experimental Results

In this section, we present simulation results to demonstrate the performance of the algorithms discussed in Section 3. We first illustrate the performance of Algorithm 2 for estimating the conditional mutual information of jointly Gaussian random variables. Next, we consider the performance of Algorithm 1 for estimating mutual information, by implementing the regulatory network inference algorithm in [18], but replacing the Gaussian kernel mutual information estimator employed there with Algorithm 1. Finally, we compare the network inference performance of Algorithm 3 with that of ARACNE [18] and BANJO [11] on synthetic networks.

### 4.1. Conditional Mutual Information of Jointly Gaussian Random Variables

To assess the accuracy of Algorithms 1 and 2 for the estimation of gene regulatory networks, we consider estimating the pairwise and conditional mutual information of multivariate Gaussian distributions. In our simulation, we compare the MI and CMI estimates of Algorithms 1 and 2 with those of the b-spline estimators. A b-spline MI estimator is proposed in [34] which divides the sample range into a number of bins. Contrary to the approach in the classical histogram estimators, where each sample contributes only to the bin it is in, for the b-spline estimator, the weight of a sample is spread to the bins. In the case of a third-order b-spline estimator, for a sample located in bin , the sample is assigned to the bins , , and , and the weight of the sample in each bin is computed using the b-spline coefficients. Here, we modify the b-spline estimator as proposed in [34] to estimate the 3-way MI  so that the CMI can be obtained with the relationship .

For MI estimation, we generated bivariate Gaussian samples with correlation coefficients 0, 0.3, and 0.6. For each coefficient, we generated  samples, and computed the estimated MI, , for each sample size using Algorithm 1 and the third-order b-spline estimator with 10 bins proposed in [34]. Each sample size is averaged over 500 sets of samples. For a bivariate Gaussian distribution, the exact MI of  and  is given by(28)

where  is the correlation coefficient between  and .

For CMI estimation, we generated samples trivariate Gaussian distributions with the following covariance matrices:(29)

For each Gaussian distribution, we generated  samples, and computed the estimated CMI, , for each sample size, using Algorithm 2 and the modified third-order b-spline estimator with 10 bins. For each sample size , the estimated CMI is averaged over 500 sets of samples. For a trivariate Gaussian distribution, the exact CMI of  and  given  is given by(30)

where , and  are the conditional covariances of , and conditional covariance between  and , given , respectively. For a trivariate Gaussian distribution, the conditional covariance matrix between  and  given  is given by(31)

where  denotes the covariance of  and . The results of the MI estimation are given in Table 3, and the results of the CMI estimation are given in Table 4. We can see that in both the MI and CMI estimation, the adaptive algorithms have closer estimates to the analytical values for all correlation coefficients and covariance matrices, except for the MI estimation for . From both tables, we can see that as the sample size grows, the adaptive algorithms converge toward the analytical values for both MI and CMI estimation. However, this is not true for the b-spline algorithms, where in the cases of MI estimation for , and CMI estimation for covariance matrix 4, the b-spline estimators converge to incorrect values.

**Table 3:** Comparison of the estimated MI of bivariate Gaussian distribution with different correlation coefficient using Algorithm 1 and b-spline algorithm.

Correlation coefficient	Algorithm	100	200	300	400	500	Analytical
0	Adaptive	0.0080	0.0036	0.0022	0.0022	0.0015	0
	b-spline 10	0.0912	0.0443	0.0288	0.0210	0.0166	
0.3	Adaptive	0.0280	0.0287	0.0305	0.0319	0.0330	0.0472
	b-spline 10	0.1248	0.0789	0.0640	0.0562	0.0515	

0.6	Adaptive	0.1371	0.1730	0.1916	0.1999	0.2052	0.2231
	b-spline 10	0.2471	0.2029	0.1879	0.1781	0.1719	

**Table 4:** Comparison of the estimated CMI of trivariate Gaussian distribution with different covariance matrices using Algorithm 2 and the modified b-spline algorithm.

Cond. Corr.	Algorithm	100	200	300	400	500	Analytical
0	Adaptive	0.0263	0.0215	0.0171	0.0175	0.0113	0
	b-spline 10	0.1899	0.1039	0.0711	0.0536	0.0429	
	b-spline 20	0.7888	0.5592	0.4330	0.3497	0.2943	
0.1612	Adaptive	0.0310	0.0278	0.0253	0.0249	0.0187	0.0132
	b-spline 10	0.1899	0.1065	0.0759	0.0603	0.0495	

0.3035	Adaptive	0.0497	0.0510	0.0534	0.0565	0.0582	0.0483
	b-spline 10	0.2251	0.1377	0.1032	0.0855	0.0761	

0.7408	Adaptive	0.2294	0.3050	0.3234	0.3444	0.3784	0.3979
	b-spline 10	0.2773	0.2390	0.2190	0.2092	0.2029	
	b-spline 20	0.6387	0.5323	0.4719	0.4378	0.4121	

As a comparison, we performed CMI estimation of covariance matrices 1 and 4 using b-spline estimator with 20 bins. In [34], it is shown that the b-spline method has similar performance to that of the kernel density estimator (KDE), and the MI computed has the same level of significance. However, the KDE is shown to be  more computationally intensive than the b-spline method. Thus in our comparisons, we only included the results from the b-spline method. For matrix 4, the b-spline estimator now converges to the correct analytical value. However, for matrix 1, the b-spline estimator does not converge to zero as the estimator with 10 bins does. This illustrates the drawback of using the b-spline estimators for MI and CMI estimation. The accuracy of the b-spline estimators depend on the choice for its parameters. On the other hand, Algorithms 1 and 2 are nonparametric, and do not need any prior knowledge of the underlying distributions to produce good estimates.

Looking more closely at CMI estimation, for small sample size and large CMI value, Algorithm 2 has a negative bias. As the sample size increases, the bias quickly reduces. On the other hand, when the true CMI value is small, Algorithm 2 tends to overestimate. It should be noted that estimating the CMI from a finite number of samples for a distribution with zero conditional correlation coefficient will typically result in a nonzero value. Nevertheless, the estimation results are still reasonably accurate, even for only 100 samples, so that conditional independence can be easily detected.

### 4.2. Regulatory Networks with Only Direct Regulation

Next, we implemented the algorithm described in [18] by replacing the Gaussian kernel MI estimator there with Algorithm 1. The modified algorithm is then compared with the original ARACNE algorithm in [18]. The purpose of this comparison is to show that the adaptive partitioning MI estimator is a valid alternative for the Gaussian kernel estimator. Specifically, we constructed 25 synthetic regulatory networks, each with 20 one-to-one gene regulations, using NetBuilder [35]. To compare the network inference performance, we adopt the same metrics as used in [18]—recall and precision. Recall, defined as , where  is the number of true positive links and  is the number of false negative links, measures the ratio of correctly identified links out of total number of links. Precision, defined as , where  is the number of false positive links, measures the ratio of correctly predicted links out of total predicted links. The values and relationship between the two metrics change with the selected threshold value, . At low , more links will be admitted as gene interactions, potentially capturing more true links, resulting in high recall values. However, as more links are included, the number of false positives also increases, which decreases the precision. On the other hand, when  is high, only links with high interactions are admitted, and they in most cases represent true interactions between genes, thus improving the precision. However, true interactions that exhibit lower interaction are not admitted, resulting in a decrease in recall.

**Figure 5:**
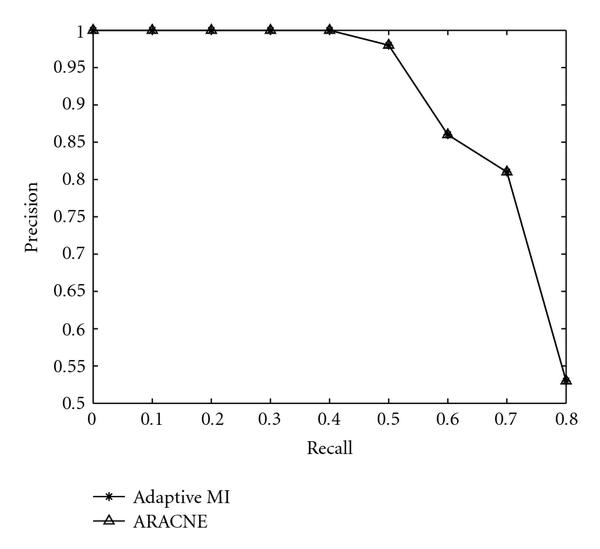
**Comparison of ARACNE and relevance-network-based algorithm with adaptive partitioning MI estimator and DPI**.

In Figure 5, we plot the precision versus recall performance of the two algorithms. It is seen that both algorithms perform exactly the same. This shows that the adaptive partitioning MI estimator can be employed as an alternative to the Gaussian kernel estimator in capturing the gene-to-gene interactions. The comparison shown in Figure 5 only uses synthetic networks constructed so that there are only pairwise connectivities. This is to illustrate that the adaptive partitioning algorithm can be used as an alternative to the kernel-based estimator in the ARACNE algorithm without degradation in performance. In the later simulations, we showed that in the presence of coregulation by two genes, the CMI is needed to improve the performance of regulatory network inference. Note that since MI and CMI are estimated from finite number of samples, the estimated MI and CMI are always greater than 0. From the relevance-network approach, by setting an arbitrarily low threshold, any number of links can be admitted as detected gene interactions, and with sufficiently low threshold, all possible links can be admitted. When large numbers of links are admitted, the number of false negative will be small, which leads to large values of recall. Thus, the comparisons at large recall values tend to be meaningless and are not included in the figures.

### 4.3. Regulatory Networks with Coregulation and Interactive Regulation

**Figure 6:**
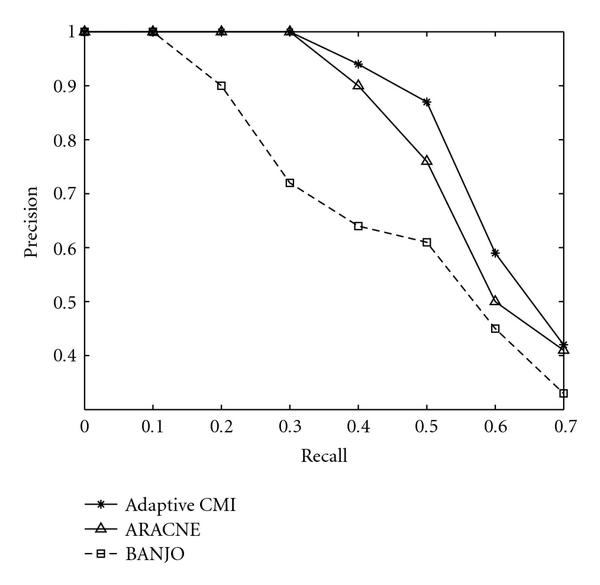
**Precision versus recall for datasets with 30% coregulated or interactively regulated links**.

**Figure 7:**
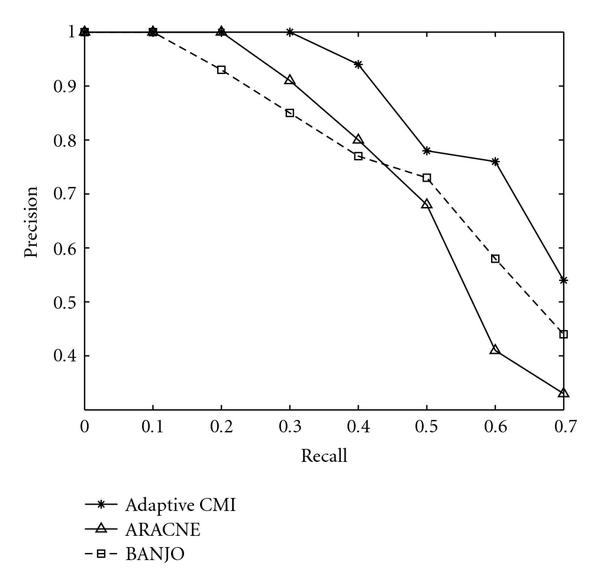
**Precision versus recall for datasets with 60% coregulated or interactively regulated links**.

We now compare the performance of Algorithm 3, ARACNE, and BANJO for regulatory network inference in the presence of coregulated and interactively regulated genes. We again use the synthetic network modeling software NetBuilder to generate random networks. NetBuilder allows modeling of gene-to-gene interactions such as activation by transcription factor combination (AND and OR), repression (NOT), and other combinatory interactions. We generated 50 synthetic networks, each containing 15 to 25 nodes with 20 links. For each node, we generated 100 steady-state expression data samples. To compare the effects of interactive regulation and coregulation on the performance of the three algorithms, two sets of synthetic networks are constructed: one set contains 25 networks where 30% of the interactions involve interactive regulation and coregulation, the other set contains 25 networks where 60% of the interactions involve interactive regulation and coregulation. In Figures 6 and 7, we plotted precision versus recall performance for the two sets of synthetic networks. It is seen that Algorithm 3 is able to outperform both ARACNE and BANJO in terms of precision for all ranges of interest. Notice that the improvement over ARACNE is greater for dataset with 60% of coregulation and interactive regulation, which is expected since ARACNE in most cases cannot detect the XOR interactions, and the application of DPI for gene coregulation can introduce both false positives and false negatives. Surprisingly, BANJO is found to have better performance than ARACNE at high recall values for the set of networks that contains 60% coregulation and interactive regulation. In [18], it is shown that the Gaussian network algorithm performs worse when the network contains only direct interaction between two genes. It is possible that due to the use of joint distributions to model the expression values of nodes in Gaussian-network-based algorithms such as BANJO, they are able to discover some of the coregulations and interactive regulations that are not found by ARACNE.

In Figure 9, we give an example of a network discovered by each algorithm. For the MI-CMI algorithm, we randomly permute for each gene the expressions across the different conditions, similar to what is done in [17]. We performed 30 such permutations, and for each permutation we computed the pairwise mutual information using Algorithm 1 for all possible pairs. The highest observed mutual information out of the 30 permutations is used as the threshold for both MI-CMI algorithm and ARACNE. Results for BANJO were obtained using the default parameters.

**Figure 8:**
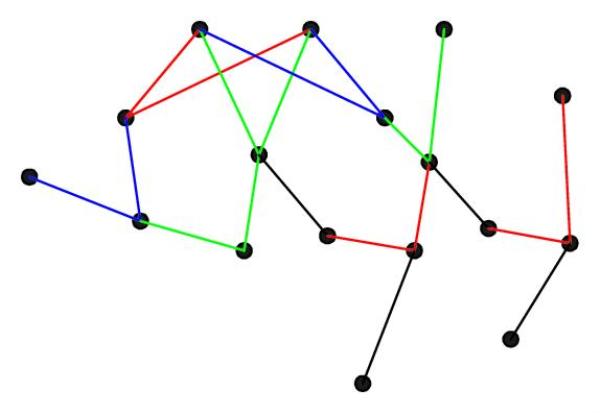
**True underlying network configuration inferred in Figure 9**.

**Figure 9:**
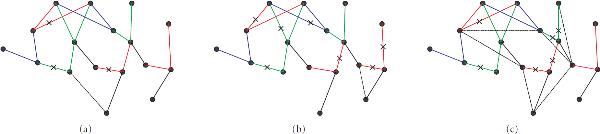
**(a) Synthetic network inferred by MI-CMI algorithm**. (b) Synthetic network inferred by ARACNE. (c) Synthetic network inferred by BANJO.

Figure 9(a) represents the network inferred by the MI-CMI algorithm, Figure 9(b) the network inferred by ARACNE, and Figure 9(c) the network discovered by BANJO. In each figure, red links represent XOR interactions, green links represent OR interactions, and blue links represent AND interactions. In Figure 9, false negative links are indicated with a cross mark, and false positive links are represented by dashed lines. The true underlying network is shown in Figure 8. As we can see from the figures, BANJO produced the most false positive links, both from indirect regulation and coregulation, whereas both the MI-CMI algorithm and ARACNE only have one each. However, the MI-CMI algorithm and BANJO discovered similar numbers of interactive regulation completely, discovering 5 and 4, respectively. An interactive regulation is completely discovered when both regulating genes are linked correctly to the interactively regulated gene. For ARACNE, only 2 interactive regulations are discovered completely, and for most of the interactive regulations only one of the links is discovered.

## 5. Conclusions

We have proposed a new gene regulatory network inference algorithm that employs both mutual information and conditional information to discover possible direct and interactive regulations between genes, and to eliminate false links due to indirect regulations and coregulation. The mutual information and conditional mutual information are estimated from the expression data using an adaptive partitioning estimator. We have shown that the proposed network inference method outperforms BANJO and ARACNE when the underlying regulatory network contains coregulated or interactively regulated genes. In this work, we have focused on the discovery of the joint regulation of a gene by two other genes. It is possible to extend this work to joint regulation by multiple genes by modifying the proposed conditional mutual information estimator to a higher order. However, doing so would pose several computational problems. As the dimension of the CMI increases, increasing number of samples is needed to maintain the same level of accuracy. Also, as the dimension of the CMI increases, the number of sets of genes to be tested also increases, thus rendering this method impractical for brute force computation of all possible sets of genes. One possibility to reduce the amount of computations needed is to take into consideration the constraints placed on the possible connectivities from known biochemical reactions between the genes involved. This can be a future direction for research in this area.
